# The Relationship Between Menopausal Status and Depression in U.S. Women: Insights from the NHANES 2017–March 2020 Cross-Sectional Study

**DOI:** 10.62641/aep.v53i6.1998

**Published:** 2025-12-17

**Authors:** Jiaojiao Pei, Jiao Chen, Ling Liu, Mao Li

**Affiliations:** ^1^Department of Obstetrics and Gynecology, The First Affiliated Hospital of Chengdu Medical College, 610500 Chengdu, Sichuan, China; ^2^Department of Hepatobiliary Surgery, The First Affiliated Hospital of Chengdu Medical College, 610500 Chengdu, Sichuan, China; ^3^Department of Stomatology, The First Affiliated Hospital of Chengdu Medical College, 610500 Chengdu, Sichuan, China; ^4^Department of Dermatology, The First Affiliated Hospital of Chengdu Medical College, 610500 Chengdu, Sichuan, China

**Keywords:** menopause, depression, POI, physical activity, NHANES

## Abstract

**Objective:**

This study harnessed cross-sectional data from the National Health and Nutrition Examination Survey (NHANES) 2017–March 2020 to examine the relationship between menopausal status and depression among U.S. women.

**Methods:**

Data from NHANES 2017–March 2020 were used for this cross-sectional analysis. Robust statistical approaches, including univariate and multivariate logistic regression, were applied, and subgroup analyses were conducted to assess the stability of the findings.

**Results:**

Women with premature ovarian insufficiency (POI) and early menopause showed a higher likelihood of depression compared with non-menopausal women (POI: odds ratio (OR) = 1.59, 95% confidence interval (95% CI) = 1.07–2.35; Early menopause: OR = 1.71, 95% CI = 1.06–2.76). Among these groups, women whose age at last delivery was under 35 years demonstrated an ever greater vulnerability to depression (POI: OR = 1.62, 95% CI = 1.07–2.43; Early menopause: OR = 1.83, 95% CI = 1.11–3.02). In postmenopausal women, moderate-intensity activity (≥150 minutes per week) was associated with increased odds of depressive symptoms (Overall moderate-to-vigorous physical activity: OR = 1.9, 95% CI = 1.08–3.34; moderate-to-vigorous recreational activity: OR = 2.17, 95% CI = 1.06–4.44). This association was not statistically significant among postmenopausal women engaging in insufficient moderate-intensity physical activity.

**Conclusion:**

These findings support a significant association between POI and early menopause and depression in U.S. women, particularly among those whose age at last delivery was below 35 years.

## Introduction

Menopause constitutes a critical phase in the senescence of the female reproductive system [1]. This physiological transition not only marks the end of reproductive capacity but is also characterized by a progressive decline in the functionality of the hypothalamic-pituitary-ovarian axis [2]. Menopause is clinically categorized into three groups based on age at onset: primary ovarian insufficiency (POI), occurring before 40 years [3, 4]; premature menopause, occurring from 40–45 years [5, 6]; and menopause, occurring after 45 years [5]. This classification is clinically relevant, as these subtypes exhibit significant differences in depression risk. As ovarian function declines, estrogen levels decrease sharply, affecting multiple physiological systems through intricate neuroendocrine pathways, with particularly pronounced effects on the central nervous system [7]. Understanding this process is essential for elucidating the pathophysiology of menopausal depression [8].

In recent years, accumulating evidence has underscored a significant association between estrogen fluctuations and depressive symptoms in women [8]. Mood disorders and depressive symptoms observed in perimenopausal women may be linked to neuroplasticity alterations induced by declining estrogen levels [9]. At the molecular level, estrogen plays a crucial role in neuronal survival and synaptic plasticity by modulating the expression of brain-derived neurotrophic factor within the hippocampus [10]. It also contributes directly to mood regulation by influencing the serotonergic system [11]. Notably, recent epigenetic research suggests that estrogen may impact depression pathogenesis through DNA methylation and other epigenetic modifications, offering a theoretical basis for the development of novel biomarkers [12]. Neuroimaging studies further support these findings: functional magnetic resonance imaging (MRI) reveals significant changes in functional connectivity within the prefrontal cortex and limbic system, while structural MRI indicates a reduction in gray matter volume in regions critical for emotional regulation [13, 14].

However, the relationship between estrogen and depressive symptoms is more complex than previously thought. Contrary to expectations, the Danish National Cohort Study found that hormone replacement therapy may increase the risk of depression in perimenopausal women, particularly during the early phases of treatment [15]. This paradox suggests that the straightforward “estrogen deficiency theory” may be overly reductive. Recent genetic studies reveal that polymorphisms in estrogen receptor genes (such as *ERα* and *ERβ*) are associated with susceptibility to depression in females, indicating that individual genetic backgrounds may modulate estrogen’s psychological effects [16]. A Mendelian randomization analysis from the Guangzhou Biobank reported no significant association between genetically predicted estrogen levels and depressive symptoms [17], challenging traditional causal assumptions. Furthermore, longitudinal data from the Study of Women’s Health Across the Nation indicate testosterone variations may independently affect the emotional well-being in menopausal women [18]. Collectively, these findings support adopting a more comprehensive perspective on the interplay between sex hormones and mental health.

Despite advances in elucidating the biological mechanisms underlying menopausal depression, substantial gaps persist in clinical translation. Existing studies predominantly rely on hormone test data sourced from specialized medical institutions, posing challenges for implementation in primary care settings. In addition, most risk assessment models do not incorporate readily accessible clinical indicators, such as age at menopause. Most critically, there is an absence of differentiated assessment tools tailored to populations with diverse socioeconomic backgrounds and lifestyles.

Using nationally representative cross-sectional data from the National Health and Nutrition Examination Survey (NHANES) 2017–March 2020, this study systematically evaluates the relationship between menopausal status and depressive symptoms, while also examining the modifying effects of specific factors—including age at last delivery, body mass index (BMI), poverty income ratio (PIR), and physical activity during work and leisure—on this relationship. Although cross-sectional designs cannot establish causality, our stringent control for multiple confounders and comprehensive sensitivity analyses provide robust epidemiological evidence to inform the development of risk assessment tools based on clinically practical indicators.

## Methods

### Data Sources

The dataset used in this study was obtained from the NHANES, a nationally representative survey administered by the National Center for Health Statistics (NCHS) under the Centers for Disease Control and Prevention (CDC). Data were accessed and downloaded from the official NHANES website (https://wwwn.cdc.gov/nchs/nhanes/). The NHANES employs a complex, stratified, multistage probability cluster sampling design, selecting approximately 5000 noninstitutionalized U.S. individuals annually. A computer-assisted interview collected sociodemographic, health, and nutritional information, while physical examinations and biological tests were performed in Mobile Examination Centers. The dataset used in this study included information collected from 2017 to March 2020, representing the most recent available survey cycle. Data collection for the 2019–2020 cycle was halted in March 2020 due to the COVID-19 pandemic. Consequently, data from March 2019–2020 were combined with the 2017–2018 cycle.

Before data collection, the NHANES protocol was approved by the National Center for Health Statistics Institutional Review Board, and written informed consent was obtained from all participants.

### Study Population

Men and individuals under 18 years of age were excluded to focus on the relationship between menopausal status and depression. Menopausal status was determined using the NHANES Reproductive Health Questionnaire (RHD043), which asked, “What is the reason you have/SP have not had a period in the past 12 months?” Participants were first classified as premenopausal or postmenopausal based on their responses. Postmenopausal women were then categorized into three groups according to their age at menopause: POI, early menopause, and menopause. Exclusion criteria included current pregnancy, breastfeeding, recent childbirth, bilateral oophorectomy, hysterectomy, or recent usage of female hormones (e.g., estrogen or progesterone).

**Fig. 1.**
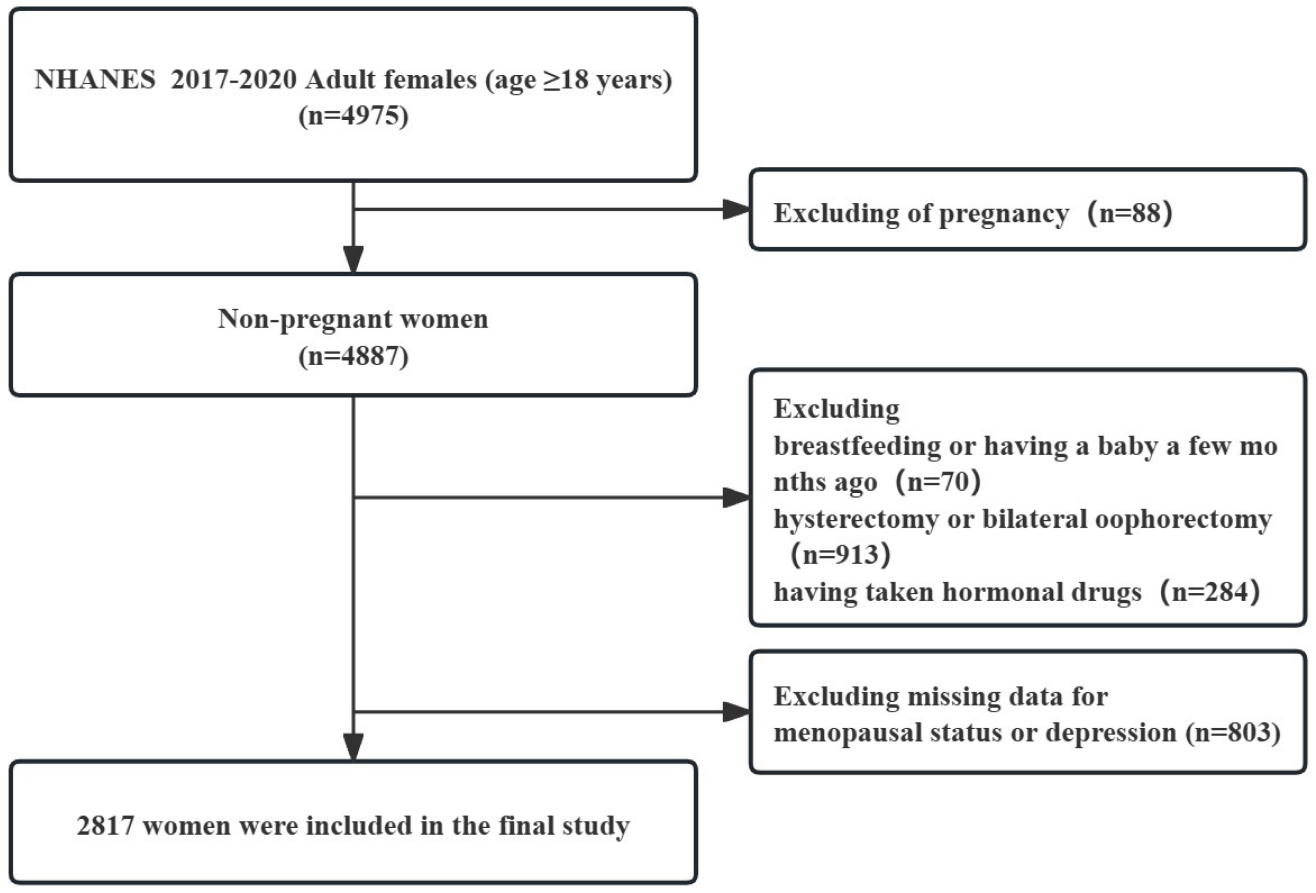
**The flow chart of the study**. NHANES, National Health and Nutrition Examination Survey.

Depression was assessed using the Patient Health Questionnaire 9 (PHQ-9), a comprehensive nine-item depression screening tool. Participants reported the frequency of depressive symptoms experienced over the previous two weeks using response options of “not at all”, “a few days”, “more than half the time”, and “almost every day”. Each item was scored from 0 to 3, yielding a total score from 0 to 27. Scores of 0–4 indicated normal mental health, while scores ≥5 indicated depression [19]. Fig. 1 presents the inclusion and exclusion criteria for the study population.

### Covariates

Covariates included demographic variables (race/ethnicity, age, education level, and PIR) and health-related factors, such as BMI, alcohol consumption, diabetes [20], hypertension, smoking status, physical activity, fertility status, and physiological indicators (hemoglobin, high-density lipoprotein cholesterol [HDL-C], total cholesterol [TC], and triglycerides [TG]).

Educational attainment was categorized as: (1) less than high school, (2) high school or equivalent, and (3) college or above. PIR was classified as low income (PIR ≤1.3), middle income (1.3 < PIR ≤ 3.5), and high income (PIR >3.5) [21]. BMI categories followed standard definitions: underweight (BMI <18.5), average weight (18.5 ≤ BMI < 25), overweight (25 ≤ BMI < 30), and obese (≥30) [22]. Alcohol consumption was categorized as never drinker, light-to-moderate drinker (females <1 standard drink/day), or heavy drinker (females ≥1 standard drink/day) [23].

Smoking status was categorized as: (1) never smoker (smoked <100 cigarettes in lifetime); (2) former smoker (smoked >100 cigarettes in lifetime but not currently smoking); and (3) current smoker (smoked >100 cigarettes in lifetime and currently smoking) [23].

Physical activity [24] was parsed into two categories: physical activity and sedentary behavior. According to the World Health Organization (WHO) guidelines on Physical Activity and Sedentary Behavior [25], individuals who participated in 150–300 minutes of moderate-intensity physical activity, 75–150 minutes of vigorous-intensity physical activity, or an equivalent combination of moderate- and vigorous-intensity activities per week were deemed to have met the recommended physical activity levels. Conversely, individuals who did not achieve these thresholds were categorized as insufficiently active.

Participants were categorized into groups such as moderate-vigorous work activity insufficient (MVWA) (<150 min/week), moderate-vigorous recreational activity short (MVRA) (<150 min/week), adequate MVWA (≥150 min/week), and adequate MVRA (≥150 minutes/week) based on self-reported data regarding the number of days and duration (in minutes) of moderate or vigorous exercise. Additionally, the threshold for insufficient walking/biking was defined as engaging in these activities for less than 150 minutes per week, as determined by self-reported data on the number of days and duration. Those failing to meet this criterion were classified as having inadequate levels of walking/biking. Furthermore, sedentary (SB) time was categorized into two groups (≥480 min/day and <480 min/day) based on self-reported estimates of daily passive sitting time [26].

### Statistical Analysis

Continuous variables with normal distribution were presented as mean ± standard deviation (SD), while skewed continuous variables were summarized as median (interquartile range, IQR). Categorical variables were expressed as frequencies and percentages. To assess differences among groups, we employed chi-square or Fisher’s exact tests for categorical variables, one-way Analysis of Variance (ANOVA) for normally distributed continuous variables, and the Kruskal–Wallis H test for skewed continuous variables.

Potential confounders were adjusted based on: (1) prior evidence in the literature; (2) statistical significance (*p* < 0.05) in univariate analyses; and (3) a substantial change (>10%) in effect size during covariate screening.

Missing data were addressed using a tailored approach. Categorical variables were handled by treating missing values as a separate category to preserve dataset integrity, while continuous variables were imputed using the k-nearest neighbors (kNN) algorithm to maintain their distributional properties, with the mean value calculated from the 10 nearest neighbors. Variables with missing data, listed in descending order of missingness, included age at first delivery (48.03%), age at last delivery (47.42%), gestation times (25.24%), parturition times (25.06%), infertility (20.4%), PIR (13.03%), HDL-C (6.67%), TG (6.96%), TC (6.60%), education level (6.57%), hypertension (6.39%), hemoglobin (4.22%), diabetes (4.15%), SB (0.75%), BMI (1.21%), age at menarche (0.67%), MVWA (0.21%), and walking/bicycling (0.21%). The overall extent of missingness ranged from 0.2% to 48.03%, with reproductive history variables showing clinically significant missingness exceeding 25%, a critical consideration given the study’s focus on menopausal status.

A multifactorial stepwise logistic regression analysis was conducted, with depression as the dependent variable and menopausal status as the primary independent variable. Both unadjusted and multivariate-adjusted models were fitted. The likelihood of depression was expressed as an odds ratio (OR) with standard error and a 95% confidence interval (95% CI). Subgroup analyses were performed stratifying for age at last delivery, BMI, PIR, MVWA, and MVRA using stratified logistic regression models. The interaction term “menopausal status × stratified variables” was included in the multivariate regression framework. Categorical variables were coded as dummy variables, and the statistical significance of the interaction terms was assessed using Wald tests. Both subgroup and interaction analyses were adjusted for potential confounders. Collinearity was assessed using the variance inflation factor, and the analysis revealed no evidence of collinearity among the covariates (see **Supplementary Table 1**).

A two-tailed alpha value of 0.05 was considered statistically significant. Analyses were performed using R software (version 4.3.1, The R Foundation for Statistical Computing, Vienna, Austria; http://www.R-project.org) and Free Statistics software (version 1.8, Beijing Fengruikelin Medical Technology Co., Ltd., Beijing, China; https://app.clinicalscientists.cn/).

## Results

### Baseline Characteristics of the Study Population

The final analysis included 2817 female participants from the NHANES 2017 to March 2020 cycle, among whom 829 reported experiencing depression, yielding a prevalence of 29.43%. Table 1 summarizes participant characteristics, including demographic factors, lifestyle behaviors, comorbidities, reproductive status, and laboratory test results. The mean age of participants was 43.6 ± 16.8 years, with subgroup-specific mean ages of 33.7 ± 10.2 years (Premenopausal), 43.2 ± 17.1 years (POI), 60.1 ± 9.9 years (Early Menopause), and 62.8 ± 8.6 years (Postmenopausal). Mean ages at menopause were 30.2 ± 7.2 years (POI), 41.6 ± 1.5 years (Early Menopause), and 50.9 ± 3.5 years (Postmenopausal).

**Table 1.** **Baseline characteristics of study patients**.

Variables			Total	Premenopause	POI	Early menopause	Postmenopause	*Statistic*	*p* value
			n = 2817	n = 1757	n = 133	n = 111	n = 816		
Age, mean (SD), y			43.6 ± 16.8	33.7 ± 10.2	43.2 ± 17.1	60.1 ± 9.9	62.8 ± 8.6	1606.177	<0.001
Race/ethnicity, n (%)								28.798	0.004
	Mexican American		368 (13.1)	248 (14.1)	18 (13.5)	16 (14.4)	86 (10.5)		
	Other Hispanic		304 (10.8)	184 (10.5)	20 (15)	11 (9.9)	89 (10.9)		
	Non-Hispanic White		880 (31.2)	517 (29.4)	49 (36.8)	40 (36)	274 (33.6)		
	Non-Hispanic Black		748 (26.6)	464 (26.4)	34 (25.6)	35 (31.5)	215 (26.3)		
	Other Race		517 (18.4)	344 (19.6)	12 (9)	9 (8.1)	152 (18.6)		
Education level, n (%)								167.841	<0.001
	Less than high school		447 (15.9)	224 (12.7)	26 (19.5)	28 (25.2)	169 (20.7)		
	High school or equivalent		584 (20.7)	305 (17.4)	48 (36.1)	32 (28.8)	199 (24.4)		
	College or above		1601 (56.8)	1054 (60)	49 (36.8)	51 (45.9)	447 (54.8)		
	NA		185 (6.6)	174 (9.9)	10 (7.5)	0 (0)	1 (0.1)		
PIR, n (%)								17.484	0.042
	≤1.3		832 (29.5)	547 (31.1)	50 (37.6)	30 (27)	205 (25.1)		
	1.3–3.5		888 (31.5)	549 (31.2)	40 (30.1)	38 (34.2)	261 (32)		
	>3.5		730 (25.9)	443 (25.2)	26 (19.5)	26 (23.4)	235 (28.8)		
	NA		367 (13.0)	218 (12.4)	17 (12.8)	17 (15.3)	115 (14.1)		
BMI, n (%), kg/m^2^								Fisher	<0.001
	<18.5		66 (2.3)	50 (2.8)	5 (3.8)	2 (1.8)	9 (1.1)		
	18.5–25		748 (26.6)	524 (29.8)	23 (17.3)	17 (15.3)	184 (22.5)		
	25–30		727 (25.8)	418 (23.8)	36 (27.1)	30 (27)	243 (29.8)		
	≥30		1242 (44.1)	746 (42.5)	67 (50.4)	58 (52.3)	371 (45.5)		
	NA		34 (1.2)	19 (1.1)	2 (1.5)	4 (3.6)	9 (1.1)		
Alcohol consumption, n (%)								189.138	<0.001
	Never drinker		396 (14.1)	214 (12.2)	20 (15)	20 (18)	142 (17.4)		
	Light-to-moderate drinker		450 (16.0)	174 (9.9)	15 (11.3)	32 (28.8)	229 (28.1)		
	Heavy drinker		1971 (70.0)	1369 (77.9)	98 (73.7)	59 (53.2)	445 (54.5)		
Hypertension, n (%)								443.765	<0.001
	No		1426 (50.6)	1130 (64.3)	64 (48.1)	29 (26.1)	203 (24.9)		
	Yes		1211 (43.0)	498 (28.3)	60 (45.1)	79 (71.2)	574 (70.3)		
	NA		180 (6.4)	129 (7.3)	9 (6.8)	3 (2.7)	39 (4.8)		
Diabetes, n (%)								Fisher	<0.001
	No		2291 (81.3)	1538 (87.5)	105 (78.9)	83 (74.8)	565 (69.2)		
	Yes		409 (14.5)	143 (8.1)	21 (15.8)	24 (21.6)	221 (27.1)		
	NA		117 (4.2)	76 (4.3)	7 (5.3)	4 (3.6)	30 (3.7)		
Smoking status, n (%)								57.941	<0.001
	Never smoker		1968 (69.9)	1287 (73.2)	84 (63.2)	67 (60.4)	530 (65)		
	Current smoker		336 (11.9)	213 (12.1)	22 (16.5)	23 (20.7)	78 (9.6)		
	Former smoker		513 (18.2)	257 (14.6)	27 (20.3)	21 (18.9)	208 (25.5)		
Physical activity									
	MVWA, n (%), min/week							Fisher	<0.001
		<150	1767 (62.7)	1042 (59.3)	81 (60.9)	78 (70.3)	566 (69.4)		
		≥150	1044 (37.1)	710 (40.4)	52 (39.1)	33 (29.7)	249 (30.5)		
		NA	6 (0.2)	5 (0.3)	0 (0)	0 (0)	1 (0.1)		
	Walking/bicycling, n (%), min/week							Fisher	0.006
		<150	2532 (89.9)	1560 (88.8)	117 (88)	96 (86.5)	759 (93)		
		≥150	279 (9.9)	194 (11)	16 (12)	14 (12.6)	55 (6.7)		
		NA	6 (0.2)	3 (0.2)	0 (0)	1 (0.9)	2 (0.2)		
	MVRA, n (%), min/week							54.676	<0.001
		<150	1890 (67.1)	1091 (62.1)	100 (75.2)	90 (81.1)	609 (74.6)		
		≥150	927 (32.9)	666 (37.9)	33 (24.8)	21 (18.9)	207 (25.4)		
	SB, n (%), min/day							Fisher	0.717
		<480	1994 (70.8)	1236 (70.3)	95 (71.4)	82 (73.9)	581 (71.2)		
		≥480	802 (28.5)	510 (29)	36 (27.1)	28 (25.2)	228 (27.9)		
		NA	21 (0.7)	11 (0.6)	2 (1.5)	1 (0.9)	7 (0.9)		
	Depression, n (%)							15.296	0.002
		Yes	829 (29.4)	492 (28)	54 (40.6)	44 (39.6)	239 (29.3)		
		No	1988 (70.6)	1265 (72)	79 (59.4)	67 (60.4)	577 (70.7)		
Fertility status									
	Infertility, n (%)							1295.629	<0.001
		Yes	238 (8.4)	185 (10.5)	10 (7.5)	8 (7.2)	35 (4.3)		
		No	2004 (71.1)	1572 (89.5)	93 (69.9)	45 (40.5)	294 (36)		
		NA	575 (20.4)	0 (0)	30 (22.6)	58 (52.3)	487 (59.7)		
Menarche age, Mean ± SD			12.6 ± 1.8	12.5 ± 1.8	12.3 ± 1.9	12.7 ± 1.8	13.1 ± 1.8	20.543	<0.001
Gestation times, Median (IQR)			3.0 (2.0, 4.0)	3.0 (2.0, 4.0)	3.0 (2.0, 4.0)	3.0 (2.0, 4.7)	3.0 (2.0, 5.0)	33.321	<0.001
Parturition times, Median (IQR)			2.1 (2.0, 3.0)	2.0 (1.7, 3.0)	2.3 (1.2, 3.1)	2.2 (2.0, 4.0)	3.0 (2.0, 4.0)	47.338	<0.001
Age of first delivery, Median (IQR)			22.0 (20.0, 25.1)	21.9 (20.0, 25.1)	21.1 (19.6, 24.4)	22.0 (20.1, 25.0)	22.0 (20.5, 25.3)	16.180	0.001
Age of last delivery, Mean ± SD			28.6 ± 5.0	28.0 ± 5.1	27.9 ± 5.1	28.9 ± 4.9	29.9 ± 4.6	15.848	<0.001
Menopausal age, Mean ± SD, y			47.4 ± 8.2		30.2 ± 7.2	41.6 ± 1.5	50.9 ± 3.5	1669.298	<0.001
Relevant physiological indicators									
	Hemoglobin (mg/dL), Mean ± SD		13.2 ± 1.3	13.0 ± 1.4	13.6 ± 1.2	13.5 ± 1.2	13.4 ± 1.2	30.571	<0.001
	HDL-C (mg/dL), Mean ± SD		57.2 ± 15.8	56.6 ± 15.4	52.6 ± 12.8	58.1 ± 14.7	59.3 ± 17.0	10.226	<0.001
	TC (mg/dL), Mean ± SD		186.3 ± 38.0	178.7 ± 33.5	181.0 ± 35.2	205.0 ± 41.7	201.1 ± 41.6	80.898	<0.001
	TG (mg/dL), Median (IQR)		99.0 (69.8, 140.0)	90.0 (63.0, 129.0)	98.2 (74.7, 140.0)	119.2 (81.5, 172.0)	115.0 (84.5, 155.0)	25.810	<0.001

Abbreviations: SD, Standard deviation; IQR, interquartile range; POI, premature ovarian insufficiency; PIR, poverty income ratio; BMI, body mass index; MVWA, moderate-to-vigorous work activity; MVRA, moderate-to-vigorous recreational activity; SB, Sedentary behavior; HDL-C, high-density lipoprotein-cholesterol; TC, total cholesterol; TG, triglycerides; NA, Not Available.

Significant differences were observed across groups in race/ethnicity, education level, PIR, BMI, alcohol consumption, hypertension, diabetes, smoking status, physical activity levels, and reproductive factors, including infertility, age at menarche, gestation and parturition times, and age at first and last delivery (all *p* < 0.05). The POI group had a higher proportion of individuals with a PIR ≤1.3 (37.59%) compared to the Postmenopausal group (25.12%). The Early menopause group had the highest percentage of women with a BMI ≥30 (52.25%), and the POI group reported more instances of heavy drinking (73.68%) than the other groups. Additionally, the POI group had a younger age at last delivery (27.9 ± 5.1 years) compared to the Postmenopausal group (29.9 ± 4.6 years).

### Association Between Menopausal Status and Depression

Depression was classified according to population-specific PHQ-9 scores, following standard diagnostic criteria. Univariate logistic regression analysis revealed statistically significant associations between depression and several factors, including education level, PIR, BMI, alcohol consumption, hypertension, diabetes, smoking status, MVWA, MVRA, menarche age, gestation times, age at first birth, menopausal age, age at last birth, HDL-C, and TG (all *p* < 0.05) (Table 2).

**Table 2.** **Association of covariates with depression risk**.

Variables		OR (95% CI)	*p* value
Age		1.001 (0.996~1.006)	0.7375
Race/ethnicity			
	Mexican American	1 (Reference)	
	Other Hispanic	1.38 (1~1.91)	0.051
	Non-Hispanic White	1.09 (0.84~1.43)	0.51
	Non-Hispanic Black	1.04 (0.79~1.36)	0.798
	Other Race	0.77 (0.57~1.04)	0.094
Education level			
	Less than high school	1 (Reference)	
	High school or equivalent	0.94 (0.73~1.22)	0.658
	College or above	0.62 (0.5~0.78)	<0.001
PIR			
	≤1.3	1 (Reference)	
	1.3–3.5	0.67 (0.55~0.82)	<0.001
	>3.5	0.43 (0.35~0.54)	<0.001
BMI			
	<18.5	1 (Reference)	
	18.5–25	0.44 (0.26~0.74)	0.002
	25–30	0.68 (0.4~1.14)	0.14
	≥30	0.84 (0.51~1.41)	0.515
Alcohol consumption			
	Never drinker	1 (Reference)	
	Light-to-moderate drinker	2.15 (1.58~2.94)	<0.001
	Heavy drinker	1.62 (1.24~2.1)	<0.001
Hypertension			
	No	1 (Reference)	
	Yes	1.28 (1.08~1.51)	0.004
Diabetes			
	No	1 (Reference)	
	Yes	1.57 (1.26~1.96)	<0.001
Smoking status			
	Never smoker	1 (Reference)	
	Current smoker	2.48 (1.96~3.15)	<0.001
	Former smoker	1.71 (1.39~2.1)	<0.001
Physical activity			
	MVWA: ≥150 vs <150	1.39 (1.18~1.64)	<0.001
	walking/bicycling: ≥150 vs <150	1.18 (0.9~1.53)	0.228
	MVRA: ≥150 vs <150	0.67 (0.56~0.8)	<0.001
	SB: ≥480 vs <480	1.19 (1~1.42)	0.053
Fertility status			
	Infertility: No vs Yes	0.79 (0.59~1.04)	0.097
	Menarche age	0.91 (0.87~0.96)	<0.001
	Gestation times	1.06 (1.02~1.11)	0.007
	Parturition times	1.02 (0.98~1.07)	0.283
	Age at first delivery	0.94 (0.92~0.96)	<0.001
	Age at last delivery	0.97 (0.96~0.99)	0.002
	Menopausal age	0.97 (0.95~0.98)	<0.001
Relevant physiological indicators			
	Hemoglobin	0.994 (0.934~1.057)	0.8392
	HDL-C	0.994 (0.989~1)	0.0336
	TC	0.999 (0.997~1.001)	0.2248
	TG	1.001 (1~1.002)	0.0171

Abbreviations: CI, confidence interval; OR, Hazard ratios; PIR, poverty income ratio; BMI, body mass index; MVWA, moderate-to-vigorous work activity; MVRA, moderate-to-vigorous recreational activity; SB, Sedentary behavior; HDL-C, high-density lipoprotein-cholesterol; TC, total cholesterol; TG, triglycerides.

The multifactorial logistic regression results, illustrating the relationship between menopausal status and depression, are presented in Table 3. The study population is categorized by menopausal age into four groups: premenopausal, POI, early menopause, and postmenopausal. In the unadjusted model (Model 1) the POI, early menopause, and postmenopausal groups showed higher odds of depression compared with the premenopausal group—by 76% (OR = 1.76, 95% CI = 1.22–2.52), 69% (OR = 1.69, 95% CI = 1.14–2.5), and 6% (OR = 1.06, 95% CI = 0.89–1.28), respectively. After stepwise adjustment for age, education level, PIR, BMI, alcohol consumption, smoking status, diabetes, hypertension, MVWA, MVRA, hemoglobin, HDL-C, TC, TG, and fertility status (menarche age, gestation times, parturition times, age at first birth, age at last delivery), a significant association between menopausal status and depression persisted (POI group: OR = 1.59, 95% CI = 1.07–2.35; Early menopause group: OR = 1.71, 95% CI = 1.06–2.76). However, in the postmenopausal group, the association with depression was not statistically significant (OR = 1.19; 95% CI: 0.86–1.64; *p* = 0.298). The odds ratios between the unadjusted and adjusted models remained stable.To account for potential confounding variables and evaluate the influence of COVID-19 and missing data on outcome measures, we utilized standardized adjustment strategies. Sensitivity analyses, incorporating pre-pandemic data from 2017 to 2018 and imputation of missing values, corroborated the robustness of the primary findings, as elaborated in **Supplementary Tables 2–4**.

**Table 3.** **Multivariate regression analysis of the association between menopausal status and depression**.

Variables	No.	n (%) with depression	Model 1		Model 2		Model 3		Model 4		Model 5	
			OR (95% CI)	*p* value	OR (95% CI)	*p* value	OR (95% CI)	*p* value	OR (95% CI)	*p* value	OR (95% CI)	*p* value
Premenopausal	1757	492 (28)	1 (Ref)		1 (Ref)		1 (Ref)		1 (Ref)		1 (Ref)	
POI	133	54 (40.6)	1.76 (1.22~2.52)	0.002	1.65 (1.13~2.41)	0.009	1.56 (1.06~2.3)	0.024	1.62 (1.1~2.39)	0.015	1.59 (1.07~2.35)	0.021
Early menopause	111	44 (39.6)	1.69 (1.14~2.5)	0.009	1.78 (1.13~2.81)	0.013	1.71 (1.07~2.73)	0.024	1.77 (1.11~2.83)	0.017	1.71 (1.06~2.76)	0.027
Postmenopausal	816	239 (29.3)	1.06 (0.89~1.28)	0.501	1.17 (0.86~1.59)	0.314	1.16 (0.85~1.6)	0.344	1.2 (0.87~1.65)	0.273	1.19 (0.86~1.64)	0.298
Trend test	2817	829 (29.4)	1.03 (0.97~1.1)	0.296	1.05 (0.95~1.16)	0.337	1.05 (0.95~1.17)	0.358	1.06 (0.95~1.18)	0.302	1.05 (0.95~1.18)	0.334

Notes: Model 1: No adjustment. Model 2: Adjusted for age + education level + PIR. Model 3: Model 2 + BMI + alcohol consumption + smoking status + diabetes + hypertension + MVWA + MVRA. Model 4: Model 3 + hemoglobin + HDL-C + TC + TG. Model 5: Model 4 + menarche age + gestation times + parturition times + age at first delivery + age at last delivery. Abbreviations: POI, premature ovarian insufficiency; Ref, reference; OR, Hazard ratios; CI, confidence interval.

### Subgroup Analysis of the Relationship Between Menopausal Status and Depression

The results of the subgroup analysis are presented in Table 4. Due to the limited sample sizes in certain BMI categories, BMI was dichotomized into non-obese (<25 kg/m^2^) and obese (≥25 kg/m^2^) for the subgroup analyses to ensure adequate statistical power while maintaining clinical relevance. age at last deliverybirth was divided into two groups (≥35 years versus <35 years) according to the widely recognized clinical definition of “advanced maternal age” in obstetrics. This standard threshold helps identify high-risk groups that warrant closer monitoring. No statistically significant interactions were observed for age at last birth, BMI, PIR, MVWA, and MVRA (all *p* for interaction >0.05).

**Table 4.** **Subgroup analyses of the association between menopausal status and depression**.

Subgroup		Menopausal status				*p* for trend	*p *for interaction
		Premenopause	POI	Early menopause	Postmenopause		
Age at last delivery (years)							0.511
	<35	1 (Ref)	1.62 (1.07~2.43)	1.83 (1.11~3.02)	1.23 (0.86~1.76)	0.308	
	≥35	1 (Ref)	1.62 (0.34~7.64)	0.4 (0.03~6.26)	1.19 (0.43~3.27)	0.765	
BMI (kg/m^2^)							0.379
	<25	1 (Ref)	0.93 (0.34~2.55)	4.18 (1.23~14.16)	1.95 (0.9~4.2)	0.117	
	≥25	1 (Ref)	1.75 (1.12~2.72)	1.46 (0.85~2.49)	1.1 (0.76~1.59)	0.652	
PIR							0.455
	≤1.3	1 (Ref)	1.92 (1.01~3.65)	1.64 (0.65~4.13)	1.26 (0.69~2.3)	0.457	
	1.3–3.5	1 (Ref)	1.77 (0.86~3.64)	2.14 (0.94~4.89)	1.12 (0.62~2)	0.809	
	>3.5	1 (Ref)	1.16 (0.44~3.04)	1.58 (0.57~4.34)	0.86 (0.43~1.72)	0.719	
MVWA (min/week)							0.192
	<150	1 (Ref)	1.55 (0.92~2.6)	0.97 (0.54~1.77)	0.9 (0.6~1.36)	0.513	
	≥150	1 (Ref)	1.53 (0.83~2.82)	5.35 (2.2~12.99)	1.9 (1.08~3.34)	0.03	
MVRA (min/week)							0.864
	<150	1 (Ref)	1.42 (0.9~2.25)	1.53 (0.89~2.63)	1 (0.69~1.45)	0.855	
	≥150	1 (Ref)	1.99 (0.89~4.44)	1.96 (0.65~5.95)	2.17 (1.06~4.44)	0.02	

Notes: Adjusted for age, education level, PIR, BMI, alcohol consumption, smoking status, diabetes, hypertension, MVWA, MVRA, hemoglobin, HDL-C, TC, TG, menarche age, gestation times, parturition times, age at first delivery, and age at last delivery. Abbreviations: Ref, reference; POI, premature ovarian insufficiency; BMI, body mass index; PIR, poverty income ratio; MVWA, moderate-to-vigorous work activity; MVRA, moderate-to-vigorous recreational activity.

In the POI group, compared with the Premenopausal group, the risk of depression was significantly higher among women with an age at last birth <35 years (OR = 1.62, 95% CI = 1.07–2.43), BMI ≥25 kg/m^2^ (OR = 1.75, 95% CI = 1.12–2.72), and PIR ≤1.3 (OR = 1.92, 95% CI = 1.01–3.65).

In the Early menopause group, compared with the Premenopausal group, women with an age at last birth <35 years (OR = 1.83, 95% CI = 1.11–3.02), BMI <25 kg/m^2^ (OR = 4.18, 95% CI = 1.23–14.16) and MVWA ≥150 min/week (OR = 5.35, 95% CI = 2.2–12.99) demonstrated significantly higher risk of depression.

In the Postmenopausal group, compared with the Premenopausal group, depression risk was significantly higher among women with MVWA ≥150 min/week (OR = 1.9, 95% CI = 1.08–3.34) and MVRA ≥150 min/week (OR = 2.17, 95% CI = 1.06–4.44, respectively).

## Discussion

This study systematically examined the epidemiological relationship between menopausal status and depression in women, using nationally representative cross-sectional data from the NHANES 2017–March 2020. The findings reveal a significant positive association between premature menopause—including POI and early menopause—and depressive symptoms. This association persisted even after adjusting for numerous potential confounders. Importantly, the relationship between early menopausal characteristics and depressive symptoms was more pronounced among women who completed childbearing before age 35, suggesting that the reproductive time window may serve as a key effect modifier. Furthermore, women engaging in ≥150 minutes of MVWA per week exhibited a higher risk of depression, whereas this association was not evident among women with insufficient MVWA. This finding contradicts the conventional notion that “exercise benefits mental health” [27]. Potential explanations may include work-related stress, adverse environmental conditions, or socioeconomic strain. Given the inherent limitations of cross-sectional designs, these results reflect associative rather than causal relationships, particularly concerning the dynamic interplay between work-related physical activity and depressive symptoms. To validate these preliminary findings and clarify the underlying temporal and biological mechanisms, future prospective cohort studies are warranted.

The observed relationship between menopausal status and depressive symptoms has been examined and partially validated in previous research. A cross-sectional study conducted by the National Institutes of Health (NIH) Clinical Research Center similarly found a significant association between POI and the lifetime risk of major depressive disorder [28]. Notably, the NIH study employed a structured clinical interview based on Statistical Manual of Mental Disorders (DSM) IV criteria, whereas the current study employed the PHQ-9 scale to assess the spectrum of depressive symptoms. This discrepancy in assessment methods may partly explain the heterogeneity observed across studies.

Two large-scale studies from South Korea provide cross-cultural references for this research. An analysis of 2232 postmenopausal women from the Korean National Health and Nutrition Examination Survey (2013–2018) identified an elevated risk of suicidal ideation among women with POI [29], consistent with the current findings linking early menopause to depressive symptoms. Additionally, a Korean national retrospective cohort study of 945,729 postmenopausal women identified that early menopause (<40 years) was associated with increased depression risk, whereas late menopause (≥55 years) exerted a protective effect [30]. While largely concordant with our results, several notable differences remain. Specifically, the Korean study focused on clinically diagnosed depression, whereas the present analysis assessed the full spectrum of depressive symptoms. This difference in focus may affect the direct comparability of effect sizes. Moreover, unlike prior work, this study did not observe a protective effect of late menopause, possibly due to differences in threshold definitions. Specifically, this study defined late menopause as occurring at or after 45 years of age, compared with 55 years or older in the Korean study. This discrepancy in criteria suggests that the influence of menopausal age on depressive symptoms may depend on reaching a critical threshold, with protective effects appearing when menopause is substantially delayed, such as at or beyond 55 years. This observation has important implications for future research: investigators examining the relationship between menopausal age and depressive symptoms should carefully define inclusion criteria and apply refined stratification strategies.

The mechanisms linking premature menopause to depression are multifactorial, involving immune, hormonal, and dietary pathways [31, 32]. During the perimenopausal period, immune cell activity is notably altered, with sex hormones playing a pivotal role in immune regulation. This interaction may represent a key pathological pathway connecting perimenopause to depression [33]. Additionally, early menopause or perimenopause can adversely affect multiple life domains, particularly occupational performance and career progression, thereby exacerbating psychological distress and increasing the risk of depression [34]. Dietary factors also warrant consideration: for instance, high consumption of full-fat dairy products has been associated with elevated risk of premenopausal depression, whereas intake of oily fish rich in omega-3 fatty acids may offer protective benefits. Such dietary components may influence depression risk indirectly via metabolic and hormonal pathways [35].

In contrast to previous studies, our research specifically examines the influence of age at last delivery on depression risk among women experiencing early menopause, including those with POI and early menopause. A key finding is that women whose last childbirth occurred before age 35 demonstrated a significantly higher risk of depression compared to those who are premenopausal. This strongly indicates that reproductive timing plays a crucial role in shaping long-term mental health outcomes. Delaying childbearing may reduce depression risk, potentially through prolonged exposure to endogenous hormones, such as the neuroprotective effects of estrogen, or through socioeconomic mechanisms, including higher educational attainment and economic stability. Conversely, among women experiencing early menopause or POI, an early age at last delivery may reflect accelerated reproductive aging and may intensify stressors such as unmet fertility aspirations or caregiving responsibilities, particularly in resource-limited contexts [36]. These results are consistent with the biopsychosocial model, which associates reproductive transitions with mental health outcomes through both biological mechanisms and environmental factors.

This study further identified that the early cessation of fertility may intensify the risk of depression through a bidirectional mechanism, wherein psychological stress resulting from infertility and early menopause substantially elevates the prevalence of depressive disorders [37, 38]. Future research should investigate whether age at last delivery affects depression risk through variables such as the duration of hormonal exposure, social support, or economic adaptation.

This study unexpectedly identified an association between early menopausal women engaging in ≥150 minutes of moderate-to-vigorous work-related physical activity per week and an elevated risk of depression. This finding stands in marked contrast to the extensively documented protective effect of physical activity on mental health reported in prior research [39]. Several factors may account for this anomalous observation. Firstly, the relatively small sample size within this subgroup (n = 52) may have limited the precision of the effect estimates, although statistical significance was maintained after multivariate adjustment. More importantly, the ability to engage in high-intensity work-related activities among older women may itself reflect underlying socioeconomic disadvantage. Women in this demographic often face greater occupational stress, have access to fewer economic resources, and possess lower educational attainment—factors that collectively represent key social determinants of depression risk. It is important to note that work- and leisure-related physical activity fundamentally differ in nature: the former is typically characterized by time constraints, limited autonomy, and high job demands, whereas the latter is more closely associated with voluntary participation and enjoyment. This qualitative distinction may help explain why the present study’s findings appear to diverge from established knowledge regarding physical activity and mental health. Additionally, the distinct endocrine changes experienced by women experiencing early menopause may alter their physiological response to work-related stress, potentially amplifying this adverse relationship. These findings underscore the need to consider the interplay between activity type, social context, and individual characteristics when evaluating the impact of physical activity on mental health, rather than focusing solely on activity duration.

Given that this study employed cross-sectional NHANES data, causal relationships cannot be inferred, and the potential for reverse causality cannot be excluded. Moreover, the self-reported nature of physical activity data introduces the possibility of recall and reporting biases, which may affect the accuracy and reliability of the results. Classification of reproductive stages—including POI, early menopause, and postmenopause—was based exclusively on self-reported questionnaire data, without biochemical verification (e.g., follicle-stimulating hormone or estradiol levels). Although the NHANES 2017–March 2020 cycle did not include hormone measurements, classifying menopausal status based on self-reported questionnaire data is consistent with standard practice in large-scale population studies on menopausal health [40, 41]. Nonetheless, this limitation could result in non-differential misclassification bias, particularly for women whose transitional menopausal status is difficult to self-identify. Furthermore, missing data for several critical variables, especially those relating to reproductive history, may have introduced additional bias. While the k-nearest neighbors (kNN) algorithm was applied to minimize data loss, it cannot entirely eliminate the uncertainty associated with incomplete observations, potentially affecting the precision and robustness of the estimated associations.

To address current research limitations, future studies should employ longitudinal designs with larger sample sizes and use objective movement-monitoring instruments, such as accelerometers, to more precisely analyze the relationship between activity intensity and depression risk across menopausal stages. Furthermore, integrating self-reported data with biochemical markers can substantially enhance the accuracy of menopausal status classification, especially during transitional phases such as POI and early perimenopause.

## Conclusion

In conclusion, this cross-sectional study demonstrates significant associations between POI, early menopause, and depression, while also highlighting complex relationships with patterns of physical activity. Given the observational nature of the NHANES data, these findings should be interpreted as indicative of population-level correlations rather than evidence of causal pathways.

## Availability of Data and Materials

In this analysis, public and de-identified data can be accessed through the CDC National Center for Health Statistics NHANES database, https://wwwn.cdc.gov/nchs/nhanes/Default.aspx.

## Supplementary Material

Supplementary material associated with this article can be found, in the online version, at https://doi.org/10.62641/aep.v53i6.1998.
