# Supplementary material for: The Relationship Between Menopausal Status and Depression in U.S. Women: Insights from the NHANES 2017–March 2020 Cross-Sectional Study
Source: Actas Esp Psiquiatr. 2025 Dec 17;53(6):1223–36. doi: 10.62641/aep.v53i6.1998 (PMC12728543; doi:10.62641/aep.v53i6.1998)
Supplement: Supplementary file 1 [file ActEsp-53-6-1223-1236-s1.zip › Supplementary Table 1.docx]

**Supplementary Table 1. Covariate Collinearity Diagnosis**

|  | **UnstandardizedCoefficients** | | **standardizedcoefficient** | | ***P*** | **Collinearitystatistics** | |
| --- | --- | --- | --- | --- | --- | --- | --- |
|  | **B** | **SE** | **β** | **t** |  | **Tolerance** | **VIF** |
| **(constant)** | 1.575 | 0.134 |  | 11.716 | 0 |  |  |
| **Age** | -0.001 | 0.001 | -0.05 | -1.371 | 0.171 | 0.253 | 3.945 |
| **Race/ethnicity** | -0.01 | 0.007 | -0.026 | -1.341 | 0.18 | 0.876 | 1.141 |
| **Education level** | -0.02 | 0.012 | -0.037 | -1.733 | 0.083 | 0.727 | 1.376 |
| **PIR** | -0.034 | 0.009 | -0.076 | -3.989 | 0 | 0.921 | 1.085 |
| **BMI** | 0.022 | 0.01 | 0.044 | 2.112 | 0.035 | 0.77 | 1.299 |
| **Alcohol consumption** | 0.014 | 0.012 | 0.023 | 1.169 | 0.243 | 0.863 | 1.159 |
| **Hypertension** | 0.012 | 0.015 | 0.016 | 0.795 | 0.427 | 0.82 | 1.22 |
| **Diabetes** | 0.038 | 0.017 | 0.042 | 2.194 | 0.028 | 0.913 | 1.095 |
| **Smoking status** | 0.049 | 0.011 | 0.085 | 4.364 | 0 | 0.887 | 1.128 |
| **MVWA** | 0.06 | 0.018 | 0.064 | 3.352 | 0.001 | 0.916 | 1.091 |
| **Walking/bicycling** | 0.018 | 0.027 | 0.012 | 0.664 | 0.507 | 0.971 | 1.029 |
| **MVRA** | -0.059 | 0.019 | -0.061 | -3.119 | 0.002 | 0.881 | 1.136 |
| **SB** | 0.054 | 0.018 | 0.056 | 2.94 | 0.003 | 0.928 | 1.078 |
| **Menopause status** | 0.017 | 0.011 | 0.05 | 1.593 | 0.111 | 0.342 | 2.921 |
| **Infertility** | -0.013 | 0.021 | -0.015 | -0.612 | 0.54 | 0.565 | 1.769 |
| **Menarche age** | -0.012 | 0.005 | -0.047 | -2.473 | 0.013 | 0.917 | 1.091 |
| **Gestation times** | 0.024 | 0.007 | 0.12 | 3.322 | 0.001 | 0.256 | 3.904 |
| **Parturition times** | -0.009 | 0.01 | -0.038 | -0.914 | 0.361 | 0.194 | 5.154 |
| **Age of first delivery** | -0.002 | 0.001 | -0.069 | -2.739 | 0.006 | 0.526 | 1.902 |
| **Age of last delivery** | -0.004 | 0.002 | -0.048 | -2.258 | 0.024 | 0.744 | 1.343 |
| **Hemoglobin** | 0 | 0.007 | 0.001 | 0.056 | 0.955 | 0.904 | 1.106 |
| **HDL-C** | 0.001 | 0.001 | 0.049 | 2.072 | 0.038 | 0.601 | 1.663 |
| **TC** | 0 | 0 | -0.028 | -1.252 | 0.211 | 0.663 | 1.508 |
| **TG** | 0 | 0 | 0.033 | 1.449 | 0.148 | 0.638 | 1.567 |

Abbreviations: B, Unstandardized Coefficient; SE, Standard Error; β, Standardized Coefficient; t, t-statistic; VIF, Variance Inflation Factor; PIR, poverty income ratio; BMI, body mass index; MVWA,moderate-to-vigorous work activity; MVRA,moderate-to-vigorous recreational activity; SB, Sedentary behavior; HDL-C,high-density lipoprotein-cholesterol; TC, total cholesterol; TG, triglyceride.
