# Supplementary material for: The Relationship Between Menopausal Status and Depression in U.S. Women: Insights from the NHANES 2017–March 2020 Cross-Sectional Study
Source: Actas Esp Psiquiatr. 2025 Dec 17;53(6):1223–36. doi: 10.62641/aep.v53i6.1998 (PMC12728543; doi:10.62641/aep.v53i6.1998)
Supplement: Supplementary file 1 [file ActEsp-53-6-1223-1236-s1.zip › Supplementary Table 2.docx]

**Supplementary Table S2. Multivariate regression analysis of the association between menopause status and depression(After excluding covariates)**

|  |  |  | **Model 1** | | **Model 2** | | **Model 3** | | **Model 4** | | **Model 5** | |
| --- | --- | --- | --- | --- | --- | --- | --- | --- | --- | --- | --- | --- |
| **Variables** | No. | n.event % | 1(Ref) |  | OR(95%CI) | *P* value | OR(95%CI) | *P* value | OR(95%CI) | *P* value | OR(95%CI) | *P* value |
| **Premenopaue** | 1757 | 492 (28) | 1.76 (1.22~2.52) | 0.002 | 1(Ref) |  | 1(Ref) |  | 1(Ref) |  | 1(Ref) |  |
| **POI** | 133 | 54 (40.6) | 1.69 (1.14~2.5) | 0.009 | 1.54 (1.06~2.23) | 0.023 | 1.48 (1.01~2.16) | 0.042 | 1.49 (1.02~2.18) | 0.038 | 1.49 (1.02~2.18) | 0.039 |
| **Early menopause** | 111 | 44 (39.6) | 1.06 (0.89~1.28) | 0.501 | 1.59 (1.06~2.38) | 0.024 | 1.49 (0.98~2.26) | 0.064 | 1.45 (0.95~2.21) | 0.084 | 1.46 (0.96~2.23) | 0.08 |
| **Postmenopause** | 816 | 239(29.3) | 1.03 (0.97~1.1) | 0.296 | 1.07 (0.88~1.3) | 0.485 | 0.99 (0.8~1.24) | 0.959 | 0.97 (0.78~1.21) | 0.788 | 1 (0.8~1.25) | 0.977 |
| **Trend.test** | 2817 | 829 (29.4) | 1(Ref) |  | 1.03 (0.97~1.1) | 0.336 | 1.01 (0.94~1.08) | 0.886 | 1 (0.93~1.07) | 0.941 | 1.01 (0.94~1.09) | 0.817 |

Note:

Model 1: no adjustment.

Model 2: adjusted for education level+PIR+BMI.

Model 3: Model 2+alcohol consumption+smoking status+diabetes+hypertension+MVWA+MVRA.

Model 4: Model 3+HDL-C+TG.

Model 5: Model 4**+**menarche age+gestation times+age of first delivery+age of last delivery.
