# Supplementary material for: The Relationship Between Menopausal Status and Depression in U.S. Women: Insights from the NHANES 2017–March 2020 Cross-Sectional Study
Source: Actas Esp Psiquiatr. 2025 Dec 17;53(6):1223–36. doi: 10.62641/aep.v53i6.1998 (PMC12728543; doi:10.62641/aep.v53i6.1998)
Supplement: Supplementary file 1 [file ActEsp-53-6-1223-1236-s1.zip › Supplementary Table 3.docx]

**Supplementary Table S3. Multivariate regression analysis of the association between menopause status and depression(2017-2018)**

|  |  |  | **Model 1** | | **Model 2** | |
| --- | --- | --- | --- | --- | --- | --- |
| **Variables** | No. | n.event % | OR(95%CI) | *P* value | OR(95%CI) | *P* value |
| **Premenopaue** | 843 | 225 (26.7) | 1(Ref) |  | 1(Ref) |  |
| **POI** | 56 | 23 (41.1) | 1.91 (1.1~3.33) | 0.022 | 1.79 (0.97~3.28) | 0.061 |
| **Early menopause** | 58 | 21 (36.2) | 1.56 (0.89~2.72) | 0.118 | 1.84 (0.91~3.73) | 0.089 |
| **Postmenopause** | 379 | 112 (29.6) | 1.15 (0.88~1.51) | 0.301 | 1.59 (0.97~2.61) | 0.066 |

Note:

Model 1: no adjustment.

Model 2: adjusted for age+education level+PIR+ BMI+alcohol consumption+-smoking status+diabetes+hypertension+MVWA+MVRA+hemoglobin+HDL-C+TC+TG

+menarche age+gestation times+parturition times+age of first delivery+age of last delivery.
