# Supplementary material for: The Relationship Between Menopausal Status and Depression in U.S. Women: Insights from the NHANES 2017–March 2020 Cross-Sectional Study
Source: Actas Esp Psiquiatr. 2025 Dec 17;53(6):1223–36. doi: 10.62641/aep.v53i6.1998 (PMC12728543; doi:10.62641/aep.v53i6.1998)
Supplement: Supplementary file 1 [file ActEsp-53-6-1223-1236-s1.zip › Supplementary Table 4.doc]

**Supplementary Table 4. Sensitivity analysis: Multivariate logistic regression of the association between menopause status and depression after exclusion of missing data**

|  |  |  | **Model 1** | | **Model 2** | |
| --- | --- | --- | --- | --- | --- | --- |
| **Variables** | No. | n.event % | OR(95%CI) | *P* value | OR(95%CI) | *P* value |
| **Premenopaue** | 1243.0 | 345 (27.8) | 1(Ref) |  | 1(Ref) |  |
| **POI** | 72.0 | 25 (34.7) | 1.38 (0.84~2.28) | 0.203 | 1.15 (0.67~1.98) | 0.604 |
| **Early menopause** | 46.0 | 19 (41.3) | 1.83 (1.01~3.34) | 0.048 | 1.91 (0.97~3.77) | 0.061 |
| **Postmenopause** | 257.0 | 80 (31.1) | 1.18 (0.88~1.58) | 0.275 | 1.28 (0.84~1.94) | 0.249 |
| **Trend.test** | 1618.0 | 469 (29) | 1.08 (0.98~1.18) | 0.118 | 1.1 (0.96~1.26) | 0.166 |

Note:

Model 1: no adjustment.

Model 2: adjusted for age+education level+PIR+ BMI+alcohol consumption+smoking status+diabetes+hypertension+MVWA+MVRA+hemoglobin+HDL-C+TC+TG+menarche age+gestation times+parturition times+age of first delivery+age of last delivery.
